# Genome-Wide Identification and Characterization of Long Non-Coding RNAs in Longissimus dorsi Skeletal Muscle of Shandong Black Cattle and Luxi Cattle

**DOI:** 10.3389/fgene.2022.849399

**Published:** 2022-05-16

**Authors:** Ruili Liu, Mingxuan Han, Xianxun Liu, Kun Yu, Xuejin Bai, Yajuan Dong

**Affiliations:** ^1^ College of Animal Science and Technology, Qingdao Agricultural University, Qingdao, China; ^2^ Laboratory of Animal Molecular Shandong Black Cattle Breeding Engineering Technology Center, College of Animal Science, Qingdao Agricultural University, Qingdao, China

**Keywords:** shandong black cattle, luxi cattle, lncRNA, longissimus dorsi, identification

## Abstract

There is an increasing understanding of the possible regulatory role of long non-coding RNAs (LncRNA). Studies on livestock have mainly focused on the regulation of cell differentiation, fat synthesis, and embryonic development. However, there has been little study of skeletal muscle of domestic animals and the potential role of lncRNA. In this study, the transcriptome numbers of longissimus muscle of different beef cattle (Shandong black catle and Luxi catlle) were used to construct muscle related lncRNAs-miRNA-mRNA interaction network through bioinformatics analysis. This is helpful to clarify the molecular mechanism of bovine muscle development, and can be used to promote animal husbandry and improve animal husbandry production. According to the screening criteria of |FC|≧2 and q < 0.05, a total of 1,415 transcripts (of which 480 were LncRNAs) were differentially expressed (*q* < 0.05) in the different breeds. Further, we found that the most differentially expressed LncRNAs were found on chromosome 9, in which the differentially expressed LncRNAs targeted 1,164 protein coding genes (*MYORG*, *Wnt4*, *PAK1*, *ADCY7,etc*) (upstream and downstream<50 Kb). In addition, Pearson’s correlation coefficients of co-expression levels indicated a potential trans regulatory relationship between the differentially expressed LncRNAs and 43844 mRNAs (r > 0.9). The identified co-expressed mRNAs (*MYORG*, *Dll1*, *EFNB2*, *SOX6*, *MYOCD*, and *MYLK*3) are related to the formation of muscle structure, and enriched in muscle system process, strained muscle cell differentiation, muscle cell development, striated muscle tissue development, calcium signaling, and AMPK signaling. Additionally, we also found that some LncRNAs (*LOC112444238*, *LOC101903367*, *LOC104975788*, *LOC112441863*, *LOC112449549*, and *LOC101907194*) may interact with miRNAs related to cattle muscle growth and development. Based on this, we constructed a LncRNAs-miRNA-mRNA interaction network as the putative basis for biological regulation in cattle skeletal muscle. Interestingly, a candidate differential LncRNA (*LOC104975788*) and a protein-coding gene (*Pax7*) contain miR-133a binding sites and binding was confirmed by luciferase reporter assay. *LOC104975788* may combined miR-133a competitively with *Pax7*, thus relieving the inhibitory effect of miR-133a on *Pax7* to regulate skeletal muscle development. These results will provide the theoretical basis for further study of LncRNA regulation and activity in different cattle breeds.

## Introduction

As an important economic trait that affects the production efficiency of beef cattle, meat production traits are a research focus in the field of beef cattle genetics and breeding. The analysis of the molecular regulation mechanisms of muscle growth can facilitate cattle breeding. Local cattle breeds in China typically present reduced growth, but increased meat quality when compared to imported cattle. These differences may be related to differences in skeletal muscle development among different breeds (Xu et al., 2018). The growth and development of beef cattle skeletal muscle can be affected by a variety of regulatory factors. Previous work mainly focused on contributions from DNA, mRNA, and miRNA, but regulatory effects of long non-coding RNA (LncRNA) on the growth and development of beef skeletal muscle remain poorly understood (Zhang et al., 2007; Dylan et al., 2008; Xu et al., 2020).

Previous studies reported contributions of LncRNA in the process of skeletal muscle proliferation and differentiation. Liu identified, mapped, and determined the global and skeletal muscle expression patterns of 7,188 LncRNAs, finding that these LncRNAs had similar open reading frames and expression levels as those of other mammalian LncRNAs (Sun et al., 2016; Yu-ying et al., 2017). Subsequently, Yue identified a highly expressed *LncRNA-YYW* in muscle tissue (Yue et al., 2017). Microarray analysis showed that *LncRNA-YYW* positively regulated the expression of growth hormone-1 and its downstream genes *AKT1* and *PIK3CD* in bovine myoblasts, and promoted myoblast proliferation (Dylan et al., 2008; Billerey et al., 2014). Cai found that lnc-ORA interacted with IGF2BP2 to inhibit the PI3K/AKT signaling pathway, thereby inhibiting muscle production and inducing skeletal muscle atrophy (Cai et al., 2021). Studies had shown that LncMD could be used as a competitive endogenous RNA (ceRNA) to compete with IGF2 and bound miR-125b to weaken its inhibitory effect on IGF2, and promoted the differentiation of bovine skeletal muscle satellite cells (Sun et al., 2016). Linc-MD1 had been shown to be a competitive endogenous RNA for miR-133 and miR-135 targets during myoblast differentiation. These transcription factors activated muscle-specific gene expression to control the time of muscle differentiation (Marcella Davide et al., 2011). Lnc-31 maintained the proliferation of myoblasts and counteracts differentiation (Dacia et al., 2018). The active regulation of Rock1 translation by lnc-31 was of great significance to the control of myogenesis (Ballarino et al., 2015). These results suggest that LncRNA may regulate the growth and development of beef skeletal muscle and meat quality. Although LncRNAs lack coding capacity, many LncRNAs act in various biological processes, serving as an additional level of genomic regulation.

The first embryo transfer calf in China was obtained from a vitrified frozen somatic cell cloned embryo, which combined Japan black cattle, Luxi cattle, and Bohai black cattle. The new generation core breeding group of this new breed, Shandong black cattle, was established by the filial generation. The new generation core breeding group hybridized with Shandong black cattle, and the second generation group of Japan black cattle (3/4) was crossed with Luxi cattle (1/4) to produce the new generation (3/4) × Bohai black cattle (1/4). Ideal black cattle and Shandong black cattle were selected from the second-generation group for cross-cross fixation. In this stage, several good lines were cross-bred with distant relatives, and then selected and the excellent lines were preserved. After four generations, this line was finally bred into a new Shandong black cattle variety matching line and used as a bull. In 2015, the Shandong black cattle variety was approved by the National Animal and Poultry Genetic Resources Committee as a new population and successfully established as a new cultivated variety in China (Lu, 2015; Liu et al., 2018).

In recent years, there has been growing research of LncRNA, but most work has mainly focused on the effects of neurologic diseases, tumors, embryonic development, and cell differentiation in human and mouse, with little research on domestic animals. The growth and development of beef cattle skeletal muscle are highly regulated processes. Although the contributions of DNA, mRNA, and miRNA to this regulation have been studied, the extent to which LncRNA regulates beef skeletal muscle growth and development remains poorly understood. To investigate the potential contribution of LncRNA, RNA samples were prepared from longissimus dorsi muscle tissues of Shandong black cattle (hybrid offspring) and Luxi Cattle (the first maternal generation). RNA-seq technology was used to identify LncRNA transcripts and their genomic characteristics, and LncRNAs differentially expressed in skeletal muscle tissues in Shandong black cattle and Luxi cattle were determined. Using these data, we constructed an interaction network of LncRNA-miRNA-mRNA and muscle development which can guide future breeding efforts.

## Materials and Methods

### Ethics Statement

The methods used in this study were performed in accordance with the guidelines of Good Experimental Practices adopted by the Institute of Animal Science (Qingdao Agricultural University, Qingdao, China). All surgical procedures involving cattle were performed according to the approved protocols of the Biological Studies Animal Care and Use Committee, Shandong Province, China (Cao et al., 2014).

### Animal and Tissue Preparation

Shandong black cattle(three)and Luxi cattle(three)were used in this study. Cattle were fed three times and received approximately equal amounts of green coarse feed and concentrate feed everyday according to standard NY5127—2002 pollution-free feeding management of beef cattle. They were raised in the same environment. At the age of 18 months, three healthy male beef cattle in each group were randomly selected for this study. The selected cattle had no scratches, scars, and scabs on their bodies, with no fat deposits in the internal organs or abdomen. No disease was found during examination, and all physiological and biochemical indexes were normal. *Longissimus dorsi* muscle samples were collected and immediately frozen in liquid nitrogen for RNA extraction. The cattle used in the experiment were euthanized as follows: first, a 1–3% sodium pentobarbital solution was prepared with physiological saline, and then intravenously injected. The injection dose is 90–135 mg/kg.

### Hematoxylin and Eosin Staining of Muscle Tissue and Fast/Slow Muscle Fiber Fluorescence Staining

In order to better observe the histological morphology of muscle, we performed HE staining and fast/slow muscle staining. Paraffin sections were prepared from muscle tissue fixed with 4% paraformaldehyde. The HE staining protocol was performed as described previously (Liu et al., 2020). Briefly, dewaxing, covering with water, Hematoxylin staining, washing with water, 5% acetic acid differentiation, eosin staining, dehydration, natural drying, sealing, and image acquisition were performed.

Tissue sections were placed in a box filled with EDTA antigen repair buffer (Purchased from Shanghai Beyotime Biotechnology Co., Ltd) (ph 8.0) for antigen repair. After natural cooling, the slides were washed in PBS (pH7.4) with three ashes of 5 minutes each. BSA was added for blocking, and then the antibody was added, followed by DAPI to stain the nucleus. An autofluorescence quenching agent was added to the slices for 5 min and the samples were then washed with running water for 10 min. After natural drying, the film was sealed with an anti-fluorescence quenching sealing agent. Finally, the slices were observed under the fluorescence microscope and images were collected.

Image-Pro Plus software was used to count the images and measure the surface area. SPSS software was used for statistical analysis to determine significant differences.

### RNA Extraction, Library Construction, and Sequencing

Total RNA samples were isolated using TRIzol reagent (Invitrogen, Carlsbad, CA, United States) according to the manufacturer’s instructions. RNA degradation and contamination were monitored on 1% agarose gels. RNA purity was checked using a NanoPhotometer^®^ spectrophotometer (IMPLEN, Los Angeles, CA, United States). RNA concentration was measured using a Qubit^®^ RNA Assay Kit in a Qubit^®^ 2.0 Fluorometer (Life Technologies, Carlsbad, CA, United States). RNA integrity was assessed using a RNA Nano 6000 Assay Kit in a Bioanalyzer 2,100 system (Agilent Technologies, Santa Clara, CA, United States). Only samples with RNA Integrity Number (RIN) scores >8 were used for sequencing, which is different from the previous studies (Liu et al., 2020). The different RIN values are due to the different requirements for RNA quality when constructing different data sets. A total of 3 μg RNA per sample was used as the input material for RNA library preparation. This study used Illumina hiseq xten sequencing platform.

### Transcriptome Assembly

The original sequencing data were processed by Fastp software (v0.23.2) (Chen et al., 2018) with parameters “-Q 20 -P90” with disjointing sequence and low-quality sequence. Clean data were obtained by removing reads containing adapters, reads containing over 10% of poly(N), and low-quality reads (>50% of the bases had Phred quality scores ≤10) from the raw data. All downstream analyses were based on the high quality clean data. The *Bos taurus* genome reference genome and gene model annotation files were downloaded from the NCBI database (CHIR_1.0, NCBI) (Mahmoudi et al., 2020). An index of the reference genome was built using Bowtie v2.0.6 (Cai et al., 2015; Cai et al., 2018) and paired-end clean reads were aligned to the reference genome using HISAT v2-2.1.0 (Yuan et al., 2019). The mapped reads from each library were assembled with Cufflinks v2.2.1 (Andersson, 2009), using Cufflinks with ‘min-frags-per-transfrag = 0’ and’–library-typefr-firststrand’, and other parameters set as default.

### LncRNA Discovery

Stringtie (Pertea et al., 2015; Kovaka et al., 2019) was used to sort reads into different classes and then generate a map for each class. Based on the length of LncRNA, and the characteristics of non-protein coding sequences, we established strict screening conditions to screen LncRNA as follows:

(1) Transcripts equal to or longer than 200 bp in length, and containing two or more exons;
(2) Transcript read coverage of at least five reads;
(3) No transcripts of known mRNA or other specific non-coding RNAs (rRNA, tRNA, snoRNA, or snRNA). This screening was based on gffcompare (http://ccb.jhu.edu/software/stringtie/gff.shtml) using the same species annotation file;
(4) According to the class in the comparison result Code information (“U”, “I”, “X”) was used to screen potential lincrna and intronic LncRNA anti-sense LncRNA Coding potential prediction screening:

To further assess if the identified transcripts are LncRNAs, a variety of coding potential analysis software was integrated, including CNCI analysis, CPC analysis, Pfam protein domain analysis, and CPAT analysis (only for animals) (Langfelder et al., 2008; Jing et al., 2010; Luo et al., 2017; Ito et al., 2018). The transcripts identified as non-coding by several methods were the final potential LncRNA dataset.

### Differential Expression Analysis

Comparison of raw counts data (Wang et al., 2010) for different genes is a very effective tool for quantitative estimation of gene expression based on RNA-Seq data. This method can eliminate the influence of gene length and sequencing quantity on the determination of gene expression levels. The calculated gene expression values allow direct comparison of gene expression differences among different varieties. Here, we used DEseq (Anders and Huber, 2010) to analyze the differential expression between the treatment group with the reference group, and selected |Log2ratio| ≥2 and q < 0.05 genes as indicative of significant differential expression. The numbers of up-regulated and down-regulated genes were also obtained.

### Prediction and Analysis of Target Genes of Differential LncRNA

LncRNA can act on target genes by Cis or Trans. Cis effect refers to LncRNA acting on adjacent target genes (Cabili et al., 2011). To predict the cis-regulatory genes of LncRNA, we screened the 50 kb of sequence upstream and downstream of LncRNA and looked for potential target genes. The coexpression relationship between LncRNA and mRNA was described by Trans. The basic principle of transaction is that the function of LncRNA has nothing to do with the position of the coding gene,but it is related to the protein-coding genes it coexpressed. According to the correlation coefficient between LncRNA and mRNA expression (correlation coefficient cor ≥ 0.9); WGCNA(Weighted correlation network analysis) was used to predict the target genes of lncRNA for cluster analysis and functional enrichment analysis of LncRNA target genes. *p*-value < 0.05 was set as the significance threshold.

### Analysis of Enrichment Pathway of GO and KEGG

GO (gene ontology) (Young et al., 2010) can be used for enrichment analysis of target genes with differential expression and Goseq (Young et al., 2010) was used to analyze the target genes of differentially expressed LncRNA. A value of *p* < 0.05 is considered as significant enrichment of differentially expressed genes.

The KEGG database (Kanehisa and Goto, 2000) describes advanced functions and utility of biological systems such as cells, organisms, and ecosystems (http://www.genome.jp/kegg/). We used KOBAS V3.0 software (Bu et al., 2021) to detect the enrichment of LncRNA target genes differentially expressed in the KEGG pathway.

### Prediction of LncRNA-miRNA-mRNA Interaction

As competing endogenous RNAs (ceRNA), LncRNA can act as a sponge to adsorb miRNA and affect mRNA expression. Therefore, to further understand the function of LncRNA in the growth and development of skeletal muscle, we used TargetScan (http://www.targetscan.org/vert_71/) miRanda ((http://www.microrna.org/microrna/home.do) and PITA qtar (https://genie.weizmann.ac.il/pubs/mir07/mir07_dyn_data.html) software to predict the differentially expressed miRNAs(Sequencing has been completed) adsorbed by LncRNA and downstream-regulated mRNA. Online miRNA binding site prediction software (RNA22: http://cbcsrv.watson.ibm.com/rna22.html and RNAhybird ()) predicted potential interaction of miRNA with LncRNA. We then constructed an LncRNA-miRNA-mRNA interaction network map using Cytoscape version 3.5.1 (Shannon et al., 2003).

### Luciferase Reporter Assay

Cells were seeded in 96-well plates at a density of 5 × 103 cells (HEK-293T) per well, 24 h before transfection. The cells were co-transfected with a mixture of 50 ng Firefly luciferase (FL) reporter vectors, 5 ng Renilla luciferase (RL) reporter vectors (pRL-TK), and miRNA mimics at the indicated concentration. The miRNA mimics were obtained from Life Technologies. After 48 h, the luciferase activity was measured with a dual luciferase reporter assay system using the psiCHECK-2 vector (Promega, Madison, WI). The *LOC104975788* and *LOC536229* (*Pax7*) sequences were separately cloned into the reporter gene vector (psiCHECK-2) to synthesize the predicted miRNA mimics and control. The potential binding target of each miRNA was cloned into the 3′UTR region of r-luciferase (hrluc), and then co-transfected with the miRNA to determine the activity of R-Luciferase. F-Luciferase (hluc +) was used as an internal reference to correct for differences in transfection efficiency between different samples. The miRNA mimics and psicheck-LOC104975788 or psicheck-LOC536229 (*Pax7*) were co-transfected into 293T cells. The expression level of reporter genes was detected using a multi-functional enzyme labeling instrument, and the miRNAs that exhibited down-regulated reporter gene expression were further screened.

### Validation of Sequencing Data by Quantitative Reverse Transcription PCR (qRT-PCR)

The reaction system (20 μl) for the RT-PCR reaction consisted of the following: 1 μl of template cDNA, 10 μl each of the upstream and downstream primers, and 5 ml (5 × 1 ml vials) of RNase-free water. The thermal cycling procedure was as follows: 94°C for 10 min, 94°C for 30 s, 60°C for 30 s, and 72°C for 40 s, with 40 cycles12. The expression of GAPDH was calculated by the 2^-△△CT^ method. Primers used for qRT-PCR as shown in Supplementary Table S1.

## Results

To investigate the potential regulatory effects of LncRNA on the growth and development of beef skeletal muscle, we analyzed samples from muscles of different breeds of beef cattle.

### Apparent Differences in Muscle Fibres in Different Breeds of Beef Cattle

**FIGURE 1:**
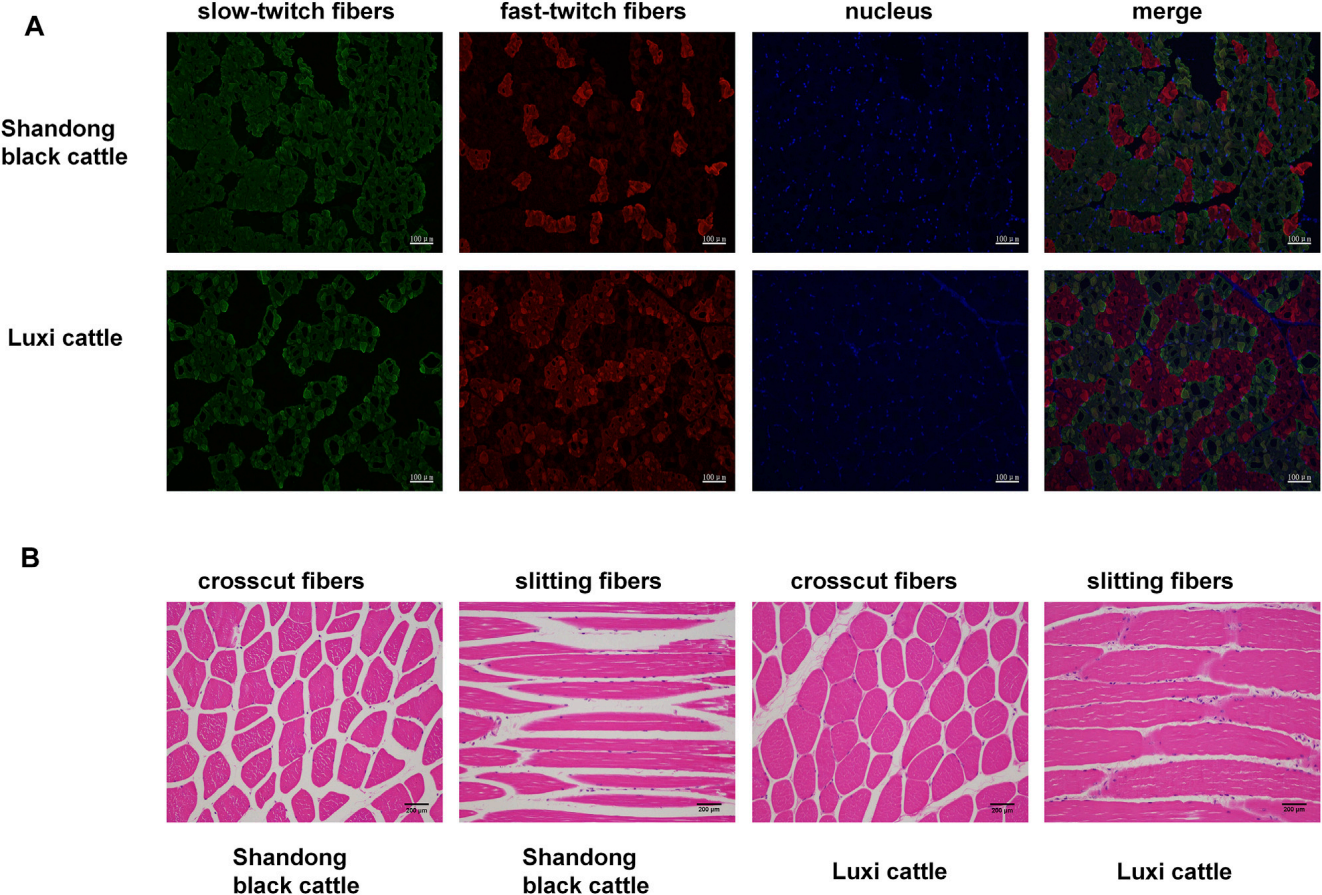
Fast/slow muscle fluorescence staining and paraffin section HE staining of muscle tissue. **(A)** Green represents slow muscle fiber, red represents fast muscle fiber, and blue represents the nucleus of the muscle cell. **(B)** Red represents a single muscle fibre, Blue represents a nucleus.

First, we obtained samples from Shandong black cattle and Luxi cattle and performed fluorescence staining sections of fast and slow muscle fibers (Figure 1A) and HE staining (Figure 1B). The results showed significant differences in muscle fibers of *longissimus dorsi* muscles between Shandong black cattle and Luxi cattle. IPP(Image-Pro Plus) software analysis showed that the average area of muscle fibres of Shandong black cattle was significantly larger than that of Luxi cattle (*p* < 0.05), with significant differences in the muscle fibre density and the ratio of fast-twitch fibers to slow-twitch fibers and slow-twitch fibers to muscle fiber area (*p* < 0.05), but not in other muscle fibre properties (*p* > 0.05) (Table 1).

**TABLE 1:** Comparison of muscle fibre characteristics and growth characteristics of different breeds of cattle.

Characteristics	Shandong black cattle	Luxi cattle
Area [μm Zhang et al. (2007)]	5,490.222 ± 184.649*	4,869.008 ± 69.596
Diameter (μm)	106.837 ± 12.537	120.491 ± 4.324
Length (μm)	174.220 ± 7.395	142.435 ± 0.968
Density (Number of muscle fibres/Muscle fiber area, EA/μm Zhang et al. (2007))	4,887.848 ± 373.586**	7,172.966 ± 319.501
Number of muscle fibres (EA)	57.667 ± 4.333	39 ± 1.732
Fast-twitch fibers/Slow-twitch fibers	0.412 ± 0.096**	3.280 ± 1.082
Fast-twitch fibers/Muscle fiber area	0.200 ± 0.0342	0.652 ± 0.110
Slow-twitch fibers/Muscle fiber area	0.508 ± 0.046*	0.246 ± 0.072
Weight (kg)	509.667 ± 2.026	489.333 ± 1.764

Note: In the table, * indicates a significant difference (*p* < 0.05); ** indicates a extremely significant difference (*p* < 0.01).

### Overview of Sequencing Data of *Longissimus Dorsi* in Beef Cattle

**FIGURE 2:**
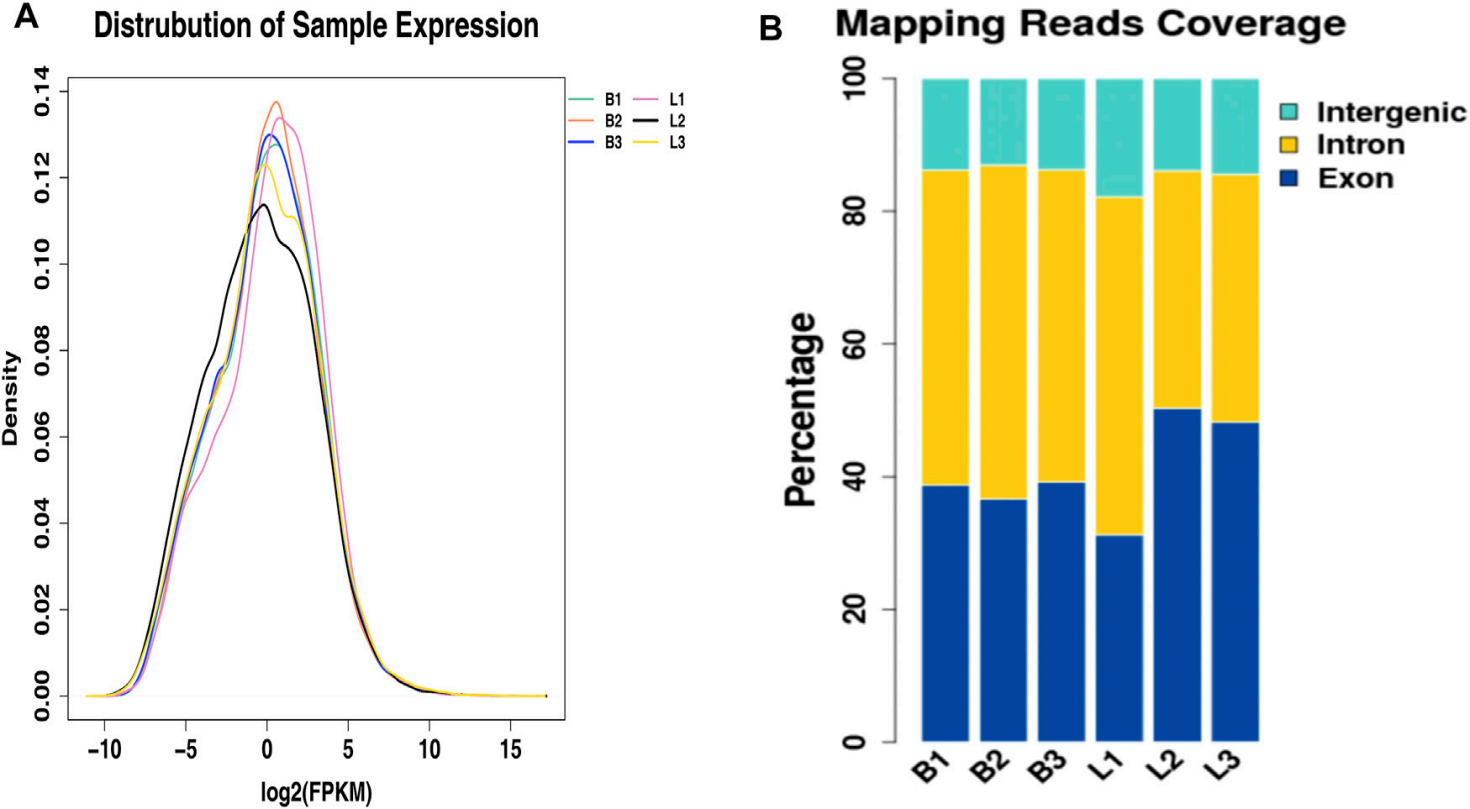
The relative expression and the genome distribution of potential transcripts and clean reads. **(A)** The new transcripts were relatively expressed in longissimus dorsi muscle of Shandong black cattle and Luxi cattle, and the density distribution map was made after taking logarithm of 2 as the base. The abscissa was log2 (FPKM +0.0001), and the ordinate was gene density. Different colors represent different samples. **(B)** The results showed that the genomic distribution of clean data of longissimus dorsi muscle tissue of Shandong black cattle and Luxi cattle was genome distribution; the abscissa was each sample, and the ordinate was the ratio of the sequence number of exon, intron and intergenic region between the reference genome.

RNA was extracted from longissimus dorsi muscle samples of Shandong black cattle and Luxi cattle and sequenced. Totals of 105345396 and 126735736 reads were obtained from Shandong black cattle and Luxi cattle, respectively, with 101145946 and 122463284 mapped reads. In the data for Luxi cattle *longissimus dorsi* muscle, the percentages of exons, introns, and genes were 43.26%, 41.34%, and 15.4%, respectively, with 38.16%, 48.3%, and 13.54%, respectively, for Shandong black cattle *longissimus dorsi* muscle tissue, as shown in Figure 2.

### Analysis of Differential mRNA Expression

A total of 20104 transcripts were obtained from the two libraries, including 18859 and 18976 mRNAs for Luxi cattle and Shandong black cattle, respectively. According to the screening criteria of |FC|≧2 and *q* < 0.05, 1,415 differentially expressed mRNA (Supplementary Table S2) were found, with 939 mRNAs significantly up-regulated and 476 down-regulated in Luxi cattle. The 92 mRNAs were specifically expressed in cattle, with 30 and 62 mRNAs specifically expressed in Shandong black cattle and Luxi Cattle, respectively (Supplementary Table S2). The top 40 genes with significant differential expression in the two breeds in cattle are listed in Supplementary Table S3, and suggest genes that could be used for the functional comparison and analysis of differences among varieties.

### Functional Analysis of Differential mRNA

**FIGURE 3:**
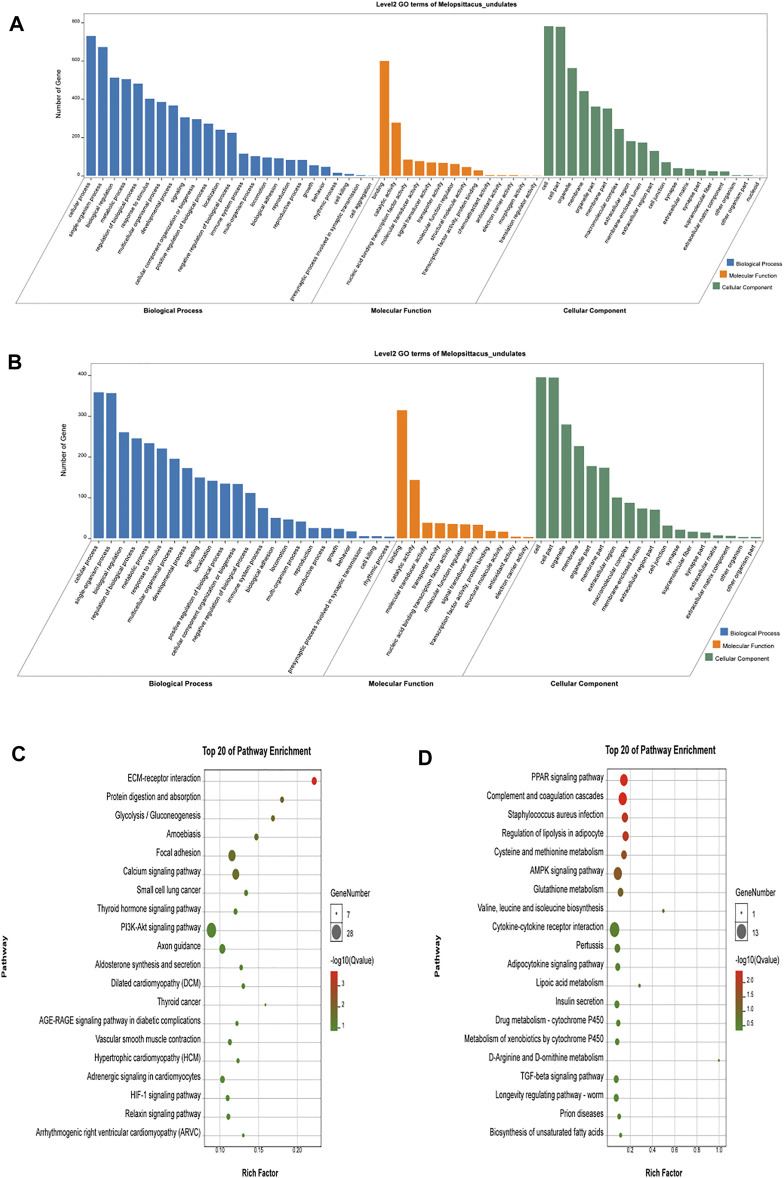
Functional analysis of differential genes. **(A,B)** The results of Go cluster enrichment analysis for up-regulated and down-regulated genes: the horizontal axis represents the name of Go entry, and the vertical axis represents the number of genes enriched in the Go entry. **(C,D)**. Up-regulated genes and down-regulated genes were analyzed by Go cluster analysis. The horizontal axis represents the name of the sample group, and the vertical axis represents the KEGG biological pathway. Each point indicates the enrichment degree of the KEGG entry, and the closer the color to green, the higher the enrichment degree. The size of each dot indicates the number of genes enriched in the KEGG entry. The larger the dot, the more genes are enriched in the KEGG entry, and vice versa.

Gene Ontology (GO) is an international standardized classification system of gene function that includes three categories: biological process (BP), molecular function (MF), and cell composition (CC). We performed GO enrichment analysis on 1,415 differentially expressed genes (Figures 3A,B), and the results showed 2,662 items (*p* < 0.05), with 388 were enriched in cell composition (CC), 361 in molecular function (MF), and 1913 in biological process (BP) in up-regulated genes (Shandong black cattle as a reference group). The enrichment results of down-regulated genes showed 1883 items in total (*p* < 0.05), with 251 enriched in cell composition (CC), 247 in molecular function (MF), and 1,385 in biological process (BP). In the muscle regulation system, muscle contraction is significantly enriched in GO terms, indicating that some differential genes may participate in muscle development and function and other life activities. Muscle-related GO terms are shown in Table 2.

**TABLE 2:** Partial GO term related muscle.

Term_type	GO Accession	*p*-value	q-value	Description	Up-regulated genes	Down-regulated genes
Biological_process	GO:0060173	0.02064757	0.211430212	Limb development. Myoblast migration. Fast muscle to slow muscle conversion. Assembly of myofibrillar cones. Hippo cascade signaling pathway. Myotube development. Fibroblast growth factor receptor signaling pathway. Notch signaling pathway. Myotube formation	*LMOD1,TNNI2,FGF10,NOTCH1,ARMH2,HOXA11,MYH3,NOTCH4,NRARP,OSR1,PODXL,WNT4,AARD, ACVRL1,ADAMTS2,ADGRG1,AGTR2,BMPER, ERRFI1,EYA1,FOXF2,GATA6,HIF1AN,HOXA10,HOXC10,HOXC11,HOXD9,HYAL2,IGFBP5,ITGA6,JAG2,LAMA5,LMOD1,MDK,MYBPC2,MYH11,MYLK3,MYOZ3,NEBL, NEURL1,NFKBIA, NOTCH3,NR4A3,OSR2,PAK1,PDPN, PEAR1,PITX1,PP3CA,PPP3R1,PRKD2,PTP4A3,RAB3A*	*AHI1,AQP2,CEBPA, CITED2,CSRP3,CXCL10,EN1,FGF9,FHOD3,FRAS1,GDF11,GSC,ID2,KLB,KRT19,LMOD2,LOC107133268,LOC11244582*3 < *MST1,MYBPH,MYH10MYOM3,MYOZ2,PAX2,PERP, POSTN,PROX1,PRR5LPRRX1,RFFL, RUNX2,SFRP5,SMAD9,SMOC1TNNI1,TP63*
	GO:0010761	0.275749234	0.592245428			
	GO:0014883	0.02191239	0.211430212			
	GO:0030239	3.42E-06	0.0003717			
	GO:0035295	0.000424469	0.016857115			
	GO:0008543	0.045842966	0.325955497			
	GO:0007219	0.009548728	0.13700442			
	GO:0035148	0.218341028	0.546196246			
	
Molecular_function	GO:0031432	0.015693382	0.275878444	Actin binding. Protomyosin binding. Protein kinase activity. Muscle structural components. Protein tyrosine kinase activity. Microtubule binding. Protein serine/threonine. Tyrosine kinase activity. Insulin like growth factor binding. Microtubule binding	*DYRK3,MYH11,PLK1,TNK2,ALK,BMX,CCDC88C,CLK1,CNTN3,EFNB2,EML1,EML6,EPHB1,KDR,KIF20A,KIF22,KIF26A,MYH1,MYH3,MYH4,MYH8,MY O 10,MY O 5B,NEBL, NTRK3,PRAG1,REEP1,RET,S100A9,STIM1,TIE1,ACVRL1,BEAN1,CAMK1D,CDKL5,EEF2K,GRK5,HTRA1,IGFBP7,IRAK2,ITGA6,LMOD1,MYBPC2,MYLK3,MYLK4,MYLPF,NEK7,NRK,PAK1,PHKA1,PIM1,PKN3,PNCK, PRKD2,PROX1,RPS6KA3,SBK2,SPEG,TPM1,TRIM63,TRPM7,TSSK1B,TSSK2,TSSK6,WISP2,WNK2*	*ANKRD2,AXL,DYNC1I1,FGF9,GAS2L2,KIF1A,KIF5C,MST1R,MYH10,MY O 1G,TRIM54,ALPK1,ANKRD1,CSRP3,FAM221A,KRT19,LIG3,LMOD2,MYBPH,MYL6B,MYOM3,NEXN, PREX2,STK32A,TSSK3,VRK2,WISP1*
	GO:0005523	0.028051936	0.362260165			
	GO:0004672	0.380205354	0.649365154			
	GO:0008307	1.45E-05	0.006860596			
	GO:0004713	0.076243247	0.395407495			
	GO:0008017	0.342175561	0.624173906			
	GO:0004712	0.656228503	0.835574106			
	GO:0005520	0.015268829	0.275878444			
	GO:0015631	0.710643065	0.863835582			
	
Cellular_component	GO:0005865	0.000202554	0.005408948	Myofibril	*TNNT2,TNNT3,TPM1,CACNA1S,FBXL22,FHL3,IGFN1,MYBPC2,MYH1,MYH3,MYH4,MYH8,MYL3,MYOZ3,NEBL,NOS1,PDE4B,PPP3CA,TRIM32,TRIM63*	*FHOD3,LMOD2,TNNI1,ANKRD1,ANKRD2,CRYAB, CSRP3,KRT19,LOC104969184,MYBPH, MYOM3,MYOZ2,NEXN, PDLIM1,SCN3B,SCN5A,SYNP O 2L,TRIM54,TWF2*

Different genes coordinate with each other to perform their biological functions in organisms. We next performed KEGG pathway analysis of the differentially expressed genes to identify the related signal transduction and metabolic pathways (Figures 3C,D). The results showed enrichment of 939 up-regulated genes in 243 pathways (*p* < 0.05), and enrichment of 476 down-regulated genes in 239 pathways, including metabolism, disease, cell communication and endocrine regulation (Supplementary Table S4). The enriched pathways (*p* < 0.05) included some signaling pathways related to skeletal muscle growth, development, and function (Table 3), such as the Wnt signaling pathway, the calcium signal transduction pathway, gap junction, the Hippo signaling pathway, the AMPK signaling pathway, and the thyroid hormone synthesis pathway.

**TABLE 3:** Partial KEGG pathway related muscle.

KEGG term (level 2)	*p*-value	q-value	UP-regulated genes	Down-regulated genes
Wnt signaling pathway	0.74905225	0.996316062	*NFATC2,NKD2,TCF7L1,CTNNB,IP1, WNT4 PPP3R1,PPP3CA*	*WNT5A,SFRP5*
Gap junction	0.8942074	0.996316062	*ADCY4,ADCY1,PDGFB*	*ADCY7*
TGF-beta signaling pathway	0.9865578	0.9998376	*LOC535280*	
Hedgehog signaling pathway	0.68703532	0.996316062	*GRK5*	*PTCH2*
FoxO signaling pathway	0.5736576	0.996316062	*S1PR1,FOX O 6,PIK3R3,GADD45B,GADD45G,PLK1*	
Hippo signaling pathway	0.5058804	0.974598156	*NKD2,TCF7L1,WNT4,TEAD1*	*WNT5A,ITGB2,DLG2,ID2,BMP8B*
Thyroid hormone synthesis	0.20608885	0.7960525	*ATP1A1,ADCY4,ADCY1,TSHR, CREB3L1*	*GPX3,ADCY7*
Ras signaling pathway	0.3897899	0.87757151	*RAPGEF5,PLA2G5,EFNA4,ANGPT2,PIK3R3,KDR,CALM3,ANGPT1,PAK1,FGF10,LOC521224,PLCE1,IGF2,PDGFB, VEGFA*	*PLA2G2D1,RASGRP1,RRAS2,FGF9,RASGRP4*
TNF signaling pathway	0.78064365	0.996317071	*NFKBIA,PIK3R3,SOCS3,CREB3L1,GR O 1*	*CXCL10,CASP3*
AMPK signaling pathway	0.4665018	0.9998376	*PFKFB2,PIK3R3,EEF2K, PFKFB3PFKM,PPARGC1A,CREB3L1*	
PI3K-Akt signaling pathway	0.959319	0.995760863	*COL6A2,COL6A1,COL4A5,COL4A6,LOC530102,COL4A2,ITGA6,COL1A1,LAMA5,NR4A1,ITGB8,COL1A2,EFNA4,ANGPT2,PIK3R3,KDR,LAMA3,DDIT4,ANGPT1,PKN3,FGF10,RXRA,COL6A3,CREB3L1,IGF2,PDGFB, VEGFA,LAMB2*	*PPP2R2C,HSP90AB1,FGF9,G6PC,PCK1*
cGMP - PKG signaling pathway	0.677879	0.992794525		*PDE3B,ADRA1A,ADORA1,ADCY7*
Rap1 signaling pathway	0.6979082	0.992794525		*PFN2,LOC101903261,RAPGEF4,FGF9,ADCY7*
Calcium signaling pathway	0.5893919	0.992794525		*RYR3,ADRA1A,PTGER3,GNA15,ADCY7*

### Screening of Differential LncRNAs

A total of 8,427 LncRNAs were found in the small RNA Library of *longissimus dorsi* muscle of Shandong black cattle and Luxi cattle. Of these, 3,498 LncRNAs were annotated in non-coding regions and the remaining 4,929 LncRNAs were in regions that are not annotated. According to the screening criteria of |FC|≧2 and *q* < 0.05, 480 differentially expressed LncRNAs were found, with 245 and 235 LncRNAs significantly up-regulated and down-regulated in Luxi Cattle compared to the levels in Shandong black cattle (Supplementary Table S5). A total of 92 LncRNAs were only expressed in one cattle breed, with 30 specific LncRNAs in Shandong black cattle and 62 specific LncRNAs in Luxi cattle (Supplementary Table S5). These breed-specific *longissimus dorsi* muscle LncRNAs included eight annotated LncRNAs in Shandong black cattle and 13 annotated LncRNAs in Luxi cattle, as shown in Table 4. The LncRNAs specifically expressed in Shandong black cattle and Luxi cattle, the first 40 unexplained LncRNAs and annotated LncRNAs are listed in Supplementary Table S6.

**TABLE 4:** LncRNA specially expressed in Shandong Black cattle and Luxi cattle.

Breed	Genes	Genomic position	*p*-value	q-value
Luxi cattle	*LOC112447810*	chrNC_037335.1:59843881-59851000	0.002587626	0.03702486
	*LOC112448752*	chrNC_037338.1:13047319-13056419	3.14E-06	0.000158716
	*LOC101905635*	chrNC_037340.1:76723459-76739979	0.000910843	0.016809347
	*LOC112449550*	chrNC_037341.1:76478493-76487345	5.40E-06	0.000252129
	*LOC104974324*	chrNC_037342.1:75777823-75780010	0.000204918	0.005162159
	*LOC104974692*	chrNC_037344.1:67130432-67132302	2.33E-05	0.000858982
	*LOC112442200*	chrNC_037345.1:45609576-45616675	0.001584248	0.025977108
	*LOC112442383*	chrNC_037345.1:62159119-62160399	0.000214833	0.005332407
	*LOC112443129*	chrNC_037348.1:25091115-25128295	0.003878832	0.049737964
	*LOC101903141*	chrNC_037353.1:47669900-47687837	7.72E-13	1.95E-10
	*LOC112444607*	chrNC_037354.1:36287211-36298269	9.39E-07	5.76E-05
	*LOC112446394*	chrNC_037331.1:68871351-68873779	1.62E-08	1.64E-06
	*LOC101907194*	chrNC_037332.1:26047494-26049444	3.59E-12	8.06E-10
Shandong Black cattle	*LOC112447868*	chrNC_037335.1:410273-419534	4.43E-06	0.000212223
	*LOC112449245*	chrNC_037340.1:3165193-3220562	7.90E-15	2.68E-12
	*LOC112441643*	chrNC_037342.1:54862373-54875026	4.23E-05	0.001397951
	*LOC112441659*	chrNC_037342.1:78782537-78785977	0.000658605	0.01318643
	*LOC104975164*	chrNC_037346.1:61277209-61278623	6.05E-05	0.001918059
	*LOC112442997*	chrNC_037347.1:23104372-23114203	0.000324072	0.007462557
	*LOC100847412*	chrNC_037348.1:32211463-32219891	1.13E-06	6.79E-05
	*LOC100847415*	chrNC_037357.1:18645452-18649324	2.19E-06	0.000118565

**FIGURE 4:**
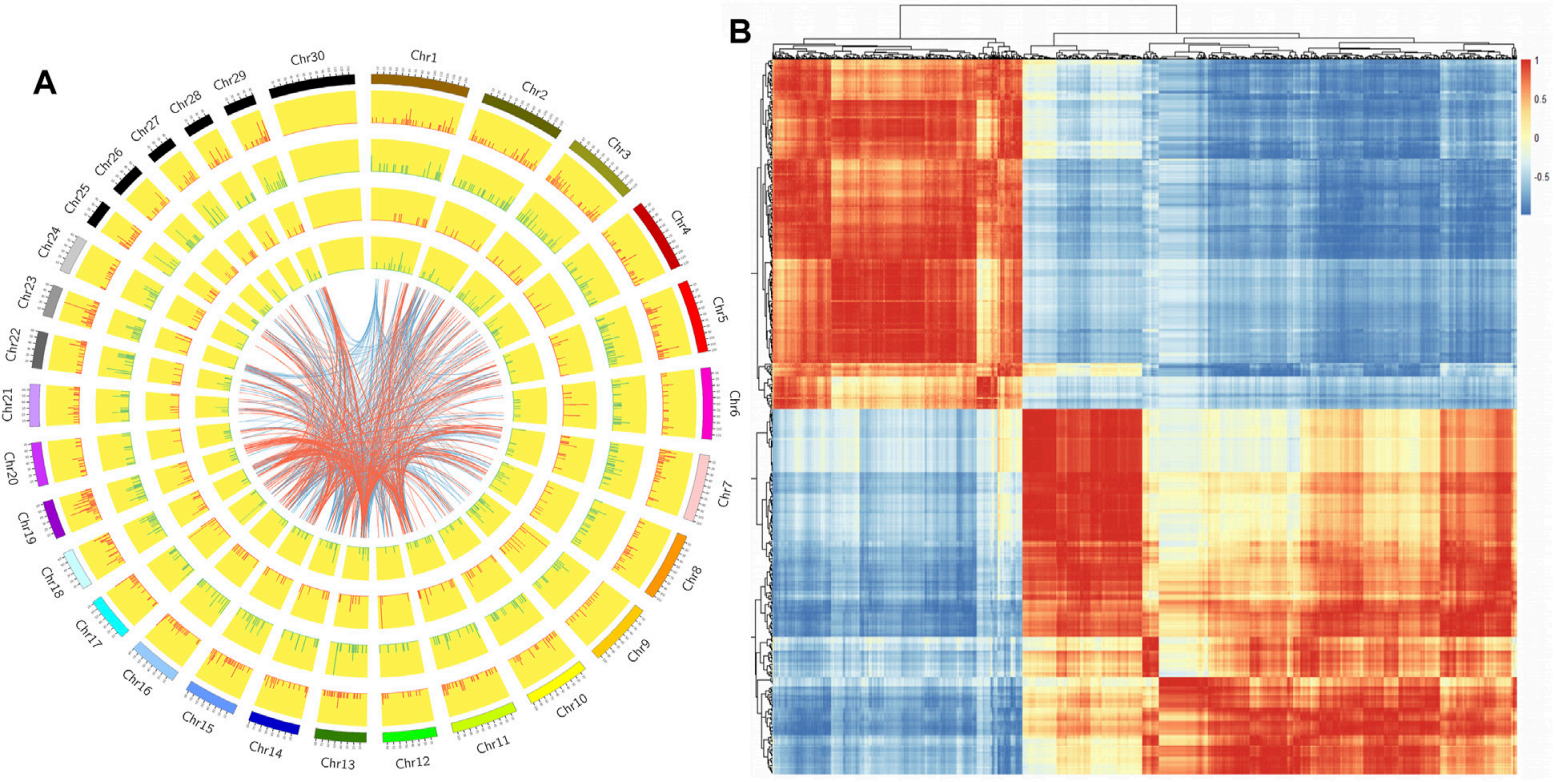
**(A)**. Circos map of differentially expressed genes and LncRNAs on cattle chromosomes. The outer ring is the diagram of autosomal distribution of the species; the first and second circles show the distribution of differentially expressed genes on chromosomes, with red lines indicating up-regulation, green lines indicating down-regulation, and the higher the column, the more differentially expressed genes in this region; the fourth and fifth circles show the distribution of LncRNAs on chromosomes, with the same expression pattern as genes. The internal lines indicate the corresponding relationship between LncRNAs and genes coexpressed by TOP 500. **(B)**. The Pearson correlation coefficient heat map of differential LncRNAs and mRNAs: Red represents positive correlation and blue represents negative correlation.

We constructed a Circos diagram to visually display the distribution of differential LncRNAs and differential mRNAs (Figure 4A). The results showed that most differentially expressed LncRNAs mapped to chromosome 9, with others mapping to chromosomes 3, 1, 11, and 7. Pearson correlation coefficient analysis of differentially expressed LncRNA and differential mRNA genes (Figure 4B) showed 43844 pairs of co-expressed LncRNAs and differential mRNA genes with correlation coefficient >0.9 (Supplementary Table S7). Based on genomic location, 387 differentially expressed LncRNAs were selected for further study.

### Prediction of Differentially Expressed LncRNAs Target Genes

LncRNAs can regulate the expression of adjacent mRNAs. We analyzed the LncRNAs and protein-coding genes by analyzing mRNAs within 50 kb of LncRNAs. The results showed that 387 of the differentially expressed LncRNAs targeted 1,164 genes. In these predictions, one LncRNA targeted multiple mRNAs and many LncRNAs targeted the same mRNA (Supplementary Table S8). Of these, *LOC112448071*, *LOC112444635*, *LOC112445963*, *LOC104975064*, *LOC101903261*, *LOC535280*, and *LOC521224* can target genes related to muscle development, including *MYORG*, *Wnt4*, *PAK1*, and *ADCY7*.

**FIGURE 5:**
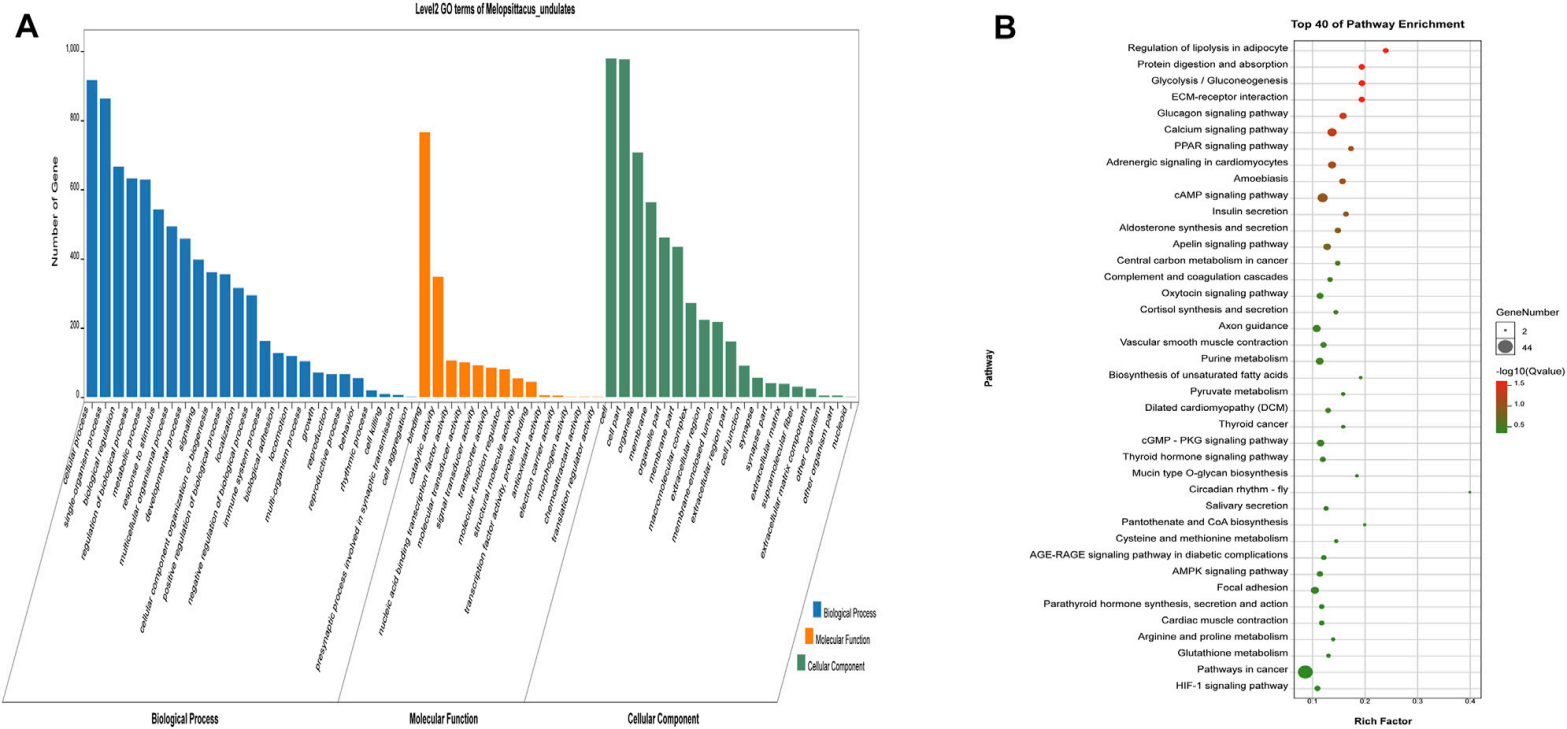
Analysis results of potential target genes GO **(A)** and KEGG **(B)** of LncRNA differentially expressed in beef cattle muscle growth and development. The legend is the same as Figure 3.

The potential target genes of LncRNA were next subjected to GO and KEGG analysis (Figure 5). We found 130 significantly enriched GO items (*p* < 0.05), with five related to the regulation of muscle development (Table 5). The 40 most enriched terms include multicellular organization development, single multicellular organization process, and multicellular organic process (Supplementary Table S9). The GO terms related to muscle development are listed in Table 5. KEGG analysis showed enrichment of 1,164 potential target genes in 299 pathways, with the 40 most significantly enriched pathways listed in Supplementary Table S9. Some of these are related to muscle development (Table 6), including the calcium signaling pathway, the AMPK signaling pathway, the cGMP-PKG signaling pathway, and the PPAR signaling pathway.

**TABLE 5:** Partial GO term related muscle.

GO term (level 2)	*p*-value	q-value	Tagert genes
Muscle structure development	4.89E-16	3.99E-13	*PITX1,MYOZ3,EPHB1,KY,MYORG, TRIM32,VGLL2,BVES, CITED2,DLL1,FHL2,LOC101903367,COL3A1,TRIM54,PLPP7,NOTCH1,RXRA,FGF9,HOXD9,CXADR, EFNB2,NEBL, GPCPD1,LAMA5,TCF15,JPH2,NFATC2,ADGRB1,CHD7,CRYAB,USP2,SOX6,MYBPH, ZBTB18,TBX1,LMOD1,TNNI1,NOS1,TBX1,MYLK3,IGFBP5,SPEG, MYBPC2,HLF,CHRND,MYH10,MYOCD,FGF10,MYOM3,WNT4,CACNA2D2,MYL3,MNF1,PIM1,JARID2,FHOD3,GATA6,MYH11,MYLPF, ANKRD1,ANKRD2,HIF1AN,NEURL1,MEF2D,CSRP3,IGF2,MBNL3,LOC112445030,S1PR1,NEXN,COL6A3,HDAC9,LMOD2,MYF5,MYL6B,PKP2,MKL1,MYOZ2,PPP3CA,UCHL1,CXCL10,SHOX2,OLFM2*
Muscle tissue development	1.11E-13	5.55E-11	*PITX1,EPHB1,MYORG, VGLL2,BVES, CITED2,DLL1,FHL2,LOC101903367,COL3A1,OSR1,ID2,NOTCH1,RXRA,FGF9,HOXD9,CXADR, EFNB2,NEBL, GPCPD1,JPH2,CHD7,EYA1,SOX6,ZBTB18,PROX1,TNNI1,TBX1,MYLK3,IGFBP5,HLF,CHRND,MYH10,MYOCD, ADAMTS9,MYL3,MNF1,PIM1,JARID2,FHOD3,GATA6,MYH11,MYLPF, ANKRD1,ANKRD2,NEURL1,MEF2D,CSRP3,S1PR1,NEXN, HDAC9,TP63,MYF5,MYL6B,PKP2,PPP3CA,SHOX2*
Striated muscle tissue development	3.06E-12	1.18E-09	*PITX1,EPHB1,MYORG, VGLL2,BVES, CITED2,DLL1,FHL2,LOC101903367,ID2,NOTCH1,RXRA,FGF9,HOXD9,CXADR, EFNB2,NEBL, GPCPD1,JPH2,CHD7,EYA1,SOX6,ZBTB18,PROX1,TNNI1,TBX1,MYLK3,HLF,CHRND,MYH10,MYOCD, ADAMTS9,MYL3,MNF1,PIM1,JARID2,FHOD3,GATA6,MYH11,MYLPF, ANKRD1,ANKRD2,NEURL1,MEF2D,CSRP3,S1PR1,NEXN, HDAC9,MYF5,MYL6B,PKP2,PPP3CA,SHOX2*
Muscle organ development	1.19E-11	4.08E-09	*PITX1,EPHB1,KY,MYORG, VGLL2,BVES, CITED2,DLL1,LOC101903367,COL3A1,NOTCH1,RXRA,FGF9,HOXD9,CXADR, EFNB2,GPCPD1,LAMA5,TCF15,JPH2,ADGRB1,CHD7,CRYAB,USP2,SOX6,ZBTB18,PROX1,TNNI1,TBX1,SPEG,HLF,CHRND, MYOCD,MYL3,MNF1,PIM1,JARID2,GATA6,MYLPF, ANKRD1,ANKRD2,NEURL1,MEF2D,CSRP3,LOC112445030,S1PR1,COL6A3,HDAC9,MYF5,MYL6B,PKP2,PPP3CA,SHOX2*
Striated muscle cell differentiation	1.88E-11	6.14E-09	*MYOZ3,MYORG, TRIM32,DLL1,FHL2,PLPP7,NOTCH1,RXRA, CXADR,EFNB2,NEBL, NFATC2,ADGRB1,SOX6,MYBPH, PROX1,LMOD1,NOS1,TBX1,MYLK3,IGFBP5,MYBPC2,MYH10,MYOCDMYOM3,CACNA2D2,FHOD3,GATA6,MYH11,ANKRD2,CSRP3,IGF2,NEXN, HDAC9,LMOD2,MYF5,MYOZ2,PPP3CA,UCHL1,CXCL10,SHOX2*

**TABLE 6:** Partial KEGG pathway related muscle.

KEGG term (level 2)	*p*-value	q-value	Tagert genes
Calcium signaling pathway	0.00083234	0.04879693	*ATP2A1,RYR3,MYLK3,P2RX6,CACNA1S,MYLK4,ADRA1A,PTGER3,PHKB, PLCD3,NOS1,CALM3,PLCD4,GNA15,ADCY4,STIM1,ORAI1,ATP2B2,HRH2,PPP3CA,ADCY1,PHKA1,ADCY7,PLCE1*
PPAR signaling pathway	0.00244591	0.0812586	*PLIN2,ME3,SCD5,FABP3,FABP7,PLIN1,RXRA, FABP4,PCK1,GK,FADS2,ADIPOQ*
Vascular smooth muscle contraction	0.02665648	0.36304747	*PPP1R14A,MYLK3,CACNA1S,MYLK4,MYH11,ADRA1A,MYH10,CALM3,ADCY4,ADCY1,MYH4,MYH1,ADCY7,MYL6B*
cGMP - PKG signaling pathway	0.02036938	0.36304747	*ATP2A1,NFATC2,MYLK3,TRPC6,CACNA1S,MYLK4,PDE3B,ADRA1A,ERI1,ATP1A1,CALM3,ADORA1,ADCY4,ATP2B2,PPP3CA,ADCY1,ADCY7,CREB3L1*
Thyroid hormone signaling pathway	0.03464076	0.38992103	*ATP2A1,ESR1,RCAN1,PLCD3,ATP1A1,WNT4,DI O 2,PLCD4,PFKM, RXRA,SLC2A1,PLCE1,NOTCH1*
AMPK signaling pathway	0.04135674	0.3978691	*PPP2R2C,SCD5,CAB39,ADRA1A,LEPR,EEF2K,PFKFB3,PFKM,FBP1,LIPE, PPARGC1A,CREB3L1,PCK1,ADIPOQ*
Cardiac muscle contraction	0.05499784	0.444442	*ATP2A1,CACNA1S,CACNA2D2,ATP1A1,MYL3,COX7A1,ACTC1,UQCRB,TPM1,LOC101902754,CACNA2D3*

### LncRNA-miRNA-mRNA Network Interaction Analysis

**FIGURE 6:**
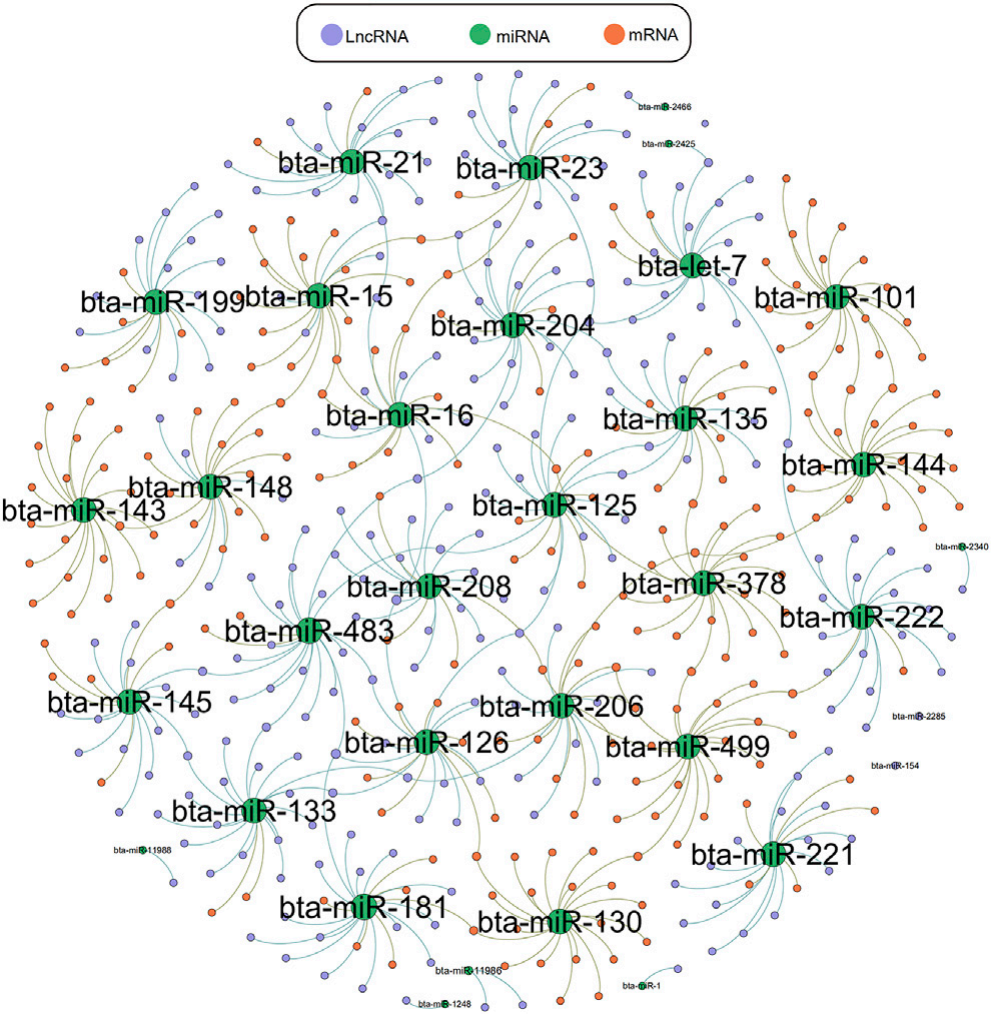
LncRNA-miRNA-mRNA network interaction diagram. LncRNA-miRNA-mRNA interaction network diagram related to muscle, green represents miRNA, orange represents mRNA, purple represents LncRNA.

We used Targetscan (Agarwal et al., 2015), Miranda (Enright et al., 2003), and PITA qtar software (Li et al., 2018) to predict the target gene relationships between mRNA and miRNA, and used Miranda and PITA qtar to predict the target gene relationships between miRNA and LncRNA. LncRNA can function as ceRNA to regulate the expression of downstream genes at the post-transcriptional level by binding miRNAs. We selected several miRNAs related to muscle development (including *miR-1*, *miR-133*, *miR-206*, *miR-181*, *miR-21*, and *miR-23a*) to predict and screen downstream-regulated mRNA using Targetscan and Miranda software. We then constructed an LncRNA-miRNA-mRNA interaction network diagram using Cytoscape version 3.5.1 (Figure 6). LncRNAs that may be involved in the development of beef cattle muscle are summarized in Table 7.

**TABLE 7:** LncRNA-miRNA-mRNA related to skeletal muscle development and growth.

LncRNA	microRNA (Targets-miR)	Targets (including potential targets)	Tissues and cells
*LOC525506,LOC526226,LOC112444238,LOC101903367*	*miR-135a/b*	*Smad5, JAK2* < *MEF2C*	C2C12, Hodgkin lymphoma
*LOC540051,LOC112448318,LOC112441863,LOC112449549,LOC101907194*	*miR-143/145*	*SRF, myocardin, Nkx2-5*	smooth muscle, skeletal muscle
*LOC112448962,LOC104975788*,*LOC112449549*	*miR-133a/b*	*SRF,KLF15,Igf1R, Runx2, dynamin 2,Pax7, MAML-1*	C2C12, skeletal muscle, cardiac muscle
*LOC112449031*	*miR-365-3p*	*SelT*	skeletal muscle
*LOC101904174*	*miR-214*	*Nras*	C2C12, skeletal muscle, cardiac muscle
*LOC104970868*	*miR-449a*	*Cdk6, Cdc25a*	C2C12 < skeletal muscle
*LOC112444607,LOC112441811, LOC101906545*	*miR-423*	*SRF, SUFU*	skeletal muscle
*LOC104975788,LOC112447073*	*miR-103*	*Wnt3a,RAI14*	C2C12 < skeletal muscle
*LOC100847415*	*miR-17-5p*	*Mfn2, RB1, ENH1*	Skeletal muscle satellite cells
*LOC525506,LOC540051,LOC514189,LOC112444238,LOC112446526,LOC112448972,LOC112446078*	*miR-21*	*PTEN, PDCD4*	skeletal muscle, cardiac muscle, smooth muscle
*LOC525506*	*miR-1*	*KCNJ2,HSP60,HSP70,caspase-9,c-Met,Pax7,Pax3,IGF-1R,KLF4*	C2C12, skeletal muscle, cardiac muscle, smoothmuscle
*LOC525506,LOC515772*	*miR-125a/b*	*IGF-II, Cbx7, SP7*	skeletal muscle, smooth muscle,ESC
*LOC112445239,LOC112441718,LOC112446120*	*miR-126*	*Spred-1, VCAM-1, IRS-1*	cardiac muscle, ESC
*LOC525506,LOC112449623*	*miR-128a*	*PPARγ, Runx1, Pax3*	skeletal muscle
*LOC112442443,LOC514189,LOC112448623,LOC100299745,LOC112443546,LOC112446047,LOC518869,LOC508468*	*miR-130a*	*GAX, HOXA5*	vascular endothelial cells (ECs)
*LOC525506,LOC112441673*	*miR-144*	*IRS1*	Type II diabetes mellitus
*LOC112441718,LOC112448623*	*miR-148a*	*ROCK1*	skeletal muscle
*LOC540051,LOC526226,LOC112444791,LOC112448967,LOC615901,LOC508180,LOC617406*	*miR-15a*	*DLK1*	3T3-L1 preadipocytes
*LOC540051,LOC112444791,LOC11244896,LOC615901,LOC508180,LOC617406*	*miR-16a/b*	*cyclin D1*	skeletal muscle
*LOC540051,LOC526226,LOC112446505,LOC112442443,LOC112444239,LOC788100*	*miR-181*	*Hox-A11 Cbx7*	skeletal muscle, ESC
*LOC525506,LOC112446505,LOC514189,LOC112446526*	*miR-199a*	*Hif-1α, Sirt1*	cardiac muscl
*LOC525506,LOC787554*	*miR-204*	*Runx2*	smooth muscle
*LOC525506,LOC112448970*	*miR-208b/499*	*Sox6, Purβ, Sp3, HP-1β*	muscle
*LOC540051,LOC514189,LOC112446078,LOC112441937*	*miR-214*	*Ezh2 N-Ras*	C2C12, skeletal muscle, ESC
*LOC112442455,LOC112446845*	*miR-221*	*p27, Mdm2*	skeletal muscle, mesenchymal cells
*LOC525506,LOC526226,LOC112442877,LOC112449155*	*miR-23a*	*MAFbx/atrogin-1, MuRF1, Myh1/2/4*	skeletal muscle

**Note:** Green represents up-regulated LncRNAs, red represents down-regulated LncRNAs, black represents LncRNAs, with no significant difference.

### Luciferase Reporter Assay

We next looked for potential miRNA binding sites of *LOC104975788* and found potential *miR-133a* and *miR-103* binding sites. Online miRNA binding site prediction software (RNA22 and RNAhybird) predicted potential interaction of *miR-133a* with *LOC104975788* and *LOC536229*(*Pax7*). The results showed that both *LOC104975788* and *LOC536229* (*Pax7*) contained *miR-133a* binding sites.

### Verification of Sequencing Results

**FIGURE 7:**
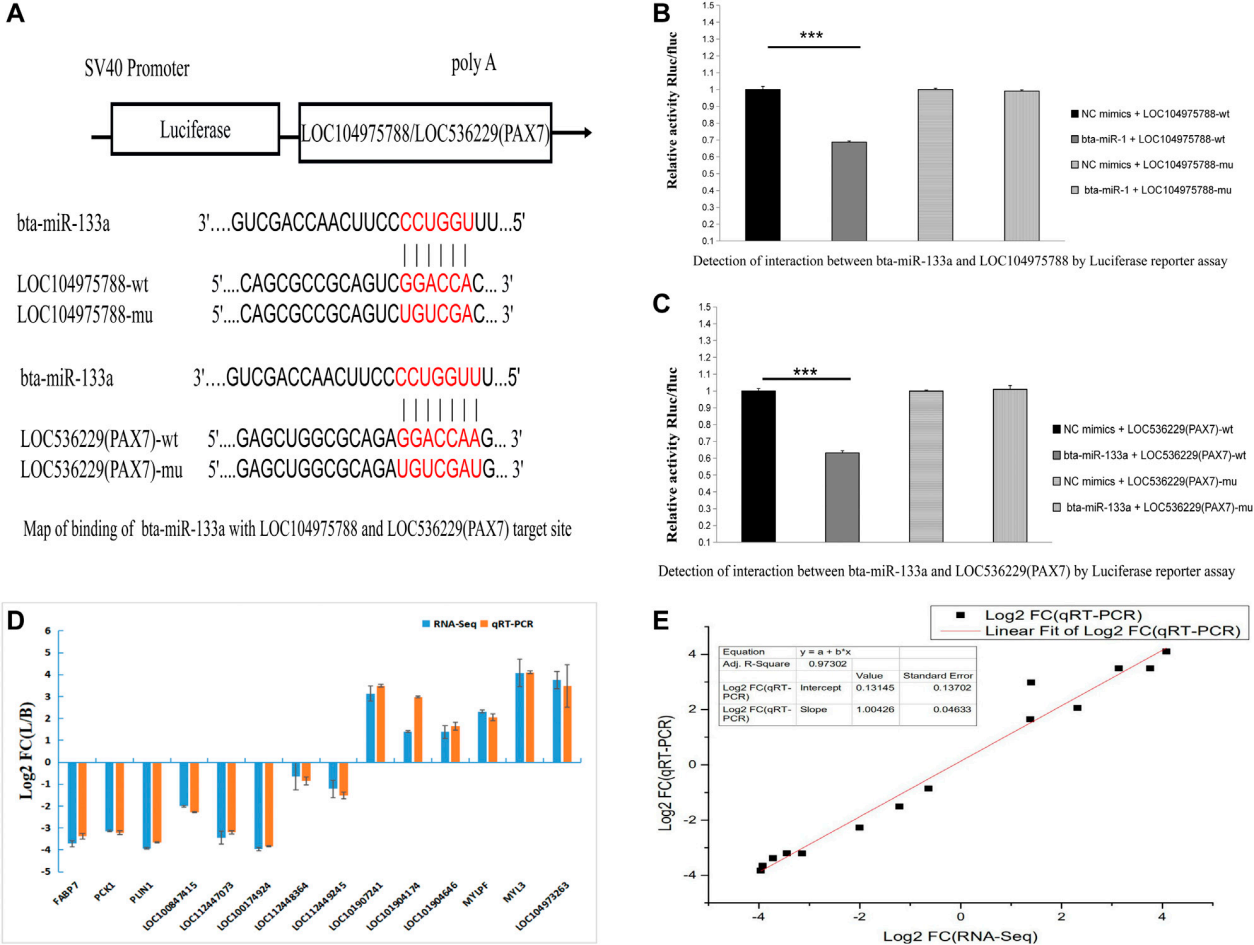
Map of binding of bta-miR-133a with LOC104975788 and LOC536229 (Pax7) target site **(A)**. Luciferase reporter assay showed significantly lower luciferase activity of miR-133a co transfected with the psi-LOC112448162 vector compared to the control group (*p* < 0.01). The luciferase activity of miR-133a co-transfected with psi-LOC536229 (Pax7) vector was also significantly decreased relative to the control Q19 group (*p* < 0.01) **(B, C)**. These results indicate that both LOC104975788 and LOC536229 (Pax7) contain miR-133a binding sites. Nine differentially expressed LncRNAs and five differentially expressed mRNAs were verified by qRT-PCR **(D, E)**.

Nine differentially expressed LncRNAs and five differentially expressed mRNAs were verified by qRT-PCR. The qRT-PCR results (Figures 7D,E) confirmed differential expression of these LncRNAs and mRNAs.

## Discussion

The muscle fiber is the basic unit of muscle, and the type of muscle fiber greatly shapes muscle characteristics (Steinbacher et al., 2006). There were obvious differences in appearance in the muscle fibres isolated from *longissimus dorsi* muscles of Shandong black cattle and Luxi cattle. The average muscle fiber area of Shandong black cattle was significantly larger than that of Luxi cattle (*p* < 0.05), and the muscle fiber diameter was smaller than that of Luxi cattle. There was a significant positive correlation between muscle fiber area and carcass traits. Muscle fiber diameter is closely related to meat quality and taste and thicker muscle fiber diameter corresponds to decreased muscle tenderness. There is a negative correlation between muscle fiber diameter and muscle fiber density (Zhou, 2010). The amount of slow muscle fiber affects sarcomere length and has an important impact on meat quality (Hendricks et al., 1971). There were significant differences in the muscle fiber density, the ratio of fast-twitch fibers/slow-twitch fibers, and weight (*p* < 0.05) in this study. These differences may be key factors leading to the differences in meat production performance and meat quality of the two breeds of cattle after birth, which was also the research basis of this study to explore the underlying molecular regulatory mechanism.

With low conservation of LncRNA sequences among species, bioinformatics methods are required to screen and identify LncRNAs. This type of analysis is based on transcript length, the number of exons, and the coding potential. In this study, transcriptome data of skeletal muscle of Shandong black cattle and Luxi cattle were generated and analyzed. In our studies, 1,415 transcripts were found to be differentially expressed, with 939 and 476 transcripts were significantly up-regulated and down-regulated in Luxi cattle (Shandong black cattle as a reference group), respectively. Further, 19 GO items and 14 regulatory pathways related to muscle development were screened by hierarchical GO and KEGG cluster analysis. Many genes involved in the regulation of muscle development were identified, including myosin protein family genes (*MYH1*, *MYH3*, *MYH4*, *MYH8*, and *MYL3*), myogenic regulatory factor family genes (*MY O 10* and *MY O 5B*), Homeobox family genes (*HOXC10*, *HOXC11*, and *HOXD9*), Troponin T family genes (*TNNT2* and *TNNT3*) and some regulatory transcription factors (*WNT4* and *TNNT2*). It is worth noting that three LncRNAs (*LOC107133268*, *LOC112445823*, *LOC104969184*) are directly enriched into muscle regulation items. Further target gene prediction analysis shows that LncRNAs may participate in the regulation of muscle development through *bta-miR-2892* (*LOC107133268*), *bta-miR-2360* and *bta-miR-2449* (*LOC112445823*). The specific regulatory mechanism needs to be further verified and analyzed.

Compared with studies in human (Federica et al., 2019) and other model organisms (James et al., 2015), there has been limited identification and characterization of beef LncRNAs, especially ones related to skeletal muscle development, with most studies of cattly focused on the identification of genes and miRNAs (Xue et al., 2013; Peters et al., 2017). In this study, we identified 8,427 multiple exon LncRNAs in beef skeletal muscle, and 480 differentially expressed LncRNAs were identified. More LncRNAs were detected in this study than previously reported in goats (Sun et al., 2013; Ying-hui et al., 2019). Fifteen randomly selected differentially expressed transcripts were verified by qPCR, and the results were consistent with the results of RNA sequencing. In conclusion, these results confirm the reliability of the identification of LncRNAs (Langfelder et al., 2008).

Although LncRNAs can act on gene sites far from their chromosomal location (Cohen et al., 2000), genes in close proximity on a chromosome may participate in the same cellular metabolic pathways and have similar biological functions (Leonardo et al., 2011). Therefore, the distribution of differentially expressed LncRNAs on chromosomes and the linkage differential expression of nearby genes may have biological significance that can help us to determine the function of a gene. The most differentially expressed LncRNAs were found on chromosome 9, followed by chromosomes 3, 1, 11, and 7. In co-expression analysis, we detected 387 differentially expressed LncRNA transcripts related to protein-coding genes according to the expression correlation coefficient values (r > 0.9), and predicted 1,164 target genes. GO analysis showed 20 GO terms related to the regulation of genes involved in muscle development. KEGG analysis showed enrichment of 1,164 potential target genes in 299 pathways, with some related to muscle development, such as the calcium signaling pathway, the AMPK signaling pathway, the cGMP - PKG signaling pathway, and the PPAR signaling pathway. Interestingly, we also found *MYORG*, *Dll1*, *EFNB2*, *SOX6*, *PROX1*, *MYOCD*, *NEBL* and *MYLK3* genes, annotated as related to muscle development. Overall, LncRNAs may play a regulatory role in skeletal muscle biological development through cis or trans mechanisms.

At the post transcriptional level, LncRNA binds miRNA competitively with mRNA for protein-coding genes, thus relieving the inhibitory effect of miRNA on protein-coding genes to promote expression of these genes (Kartha and Subramanian, 2014). We analyzed the expression patterns of LncRNAs differentially expressed among different breeds, and constructed an LncRNA-miRNA-mRNA interaction network related to skeletal muscle development. A total of 52 LncRNAs related to muscle development were identified, with nine up-regulated and five down-regulated. All of these LncRNAs contain one or more putative miRNA binding sites with 48 LncRNAs predicted to interact with one to two miRNAs related to muscle development, and other LncRNAs predicted to interact with more than three miRNAs, such as *LOC525506*, *LOC540051*, *LOC514189*. With the miRNA seed sequences, LncRNAs can bind to miRNA to act as a sponge, preventing miRNA from binding to its target mRNA. As a classic example, M. Cesana (Marcella Davide et al., 2011) confirmed that linc-MD1, a long non-coding RNA specifically expressed during myoblast differentiation, regulates the expression of muscle specific transcription regulators *MAML1* and *MEF2C* through *miR-133* and *miR-135*. In particular, *LOC525506* may be involved in the development of beef skeletal muscle by regulating *miR-1*, *miR-23a*, *miR-378*, *let-7*, *miR-483*, and *miR-21*. This is the most predicted LncRNA, but little is known about its expression and function. Both *LOC112447073* and *LOC104975788* are involved in the skeletal muscle development of beef cattle by interaction with *miR-103*. More interestingly, some target genes of *LOC112447073*, *LOC104975788*, and *LOC101903367* directly regulate the development of muscle fiber and maintain the stability and development of muscle, such as *miR-103*, *miR-133a*, *miR-145*, *MEF2C*, myocardin, and *Pax7* (Richard et al., 2008; Mathew et al., 2011). Choi identified 11 LncRNAs in bovine transcripts by studying skeletal muscle and adipose tissue of Korean cattle, with four related to muscle function (Jae et al., 2018). These data provide new insight into the role of LncRNA in muscle development and enrich the existing LncRNA mammalian skeletal muscle resources.

The second to eighth bases of the sequence at the 5 ′end of miRNA is called the seed sequence. Complete complementarity of this sequence and that of the target gene indicates the potential for binding of the target gene by miRNA (Lewis et al., 2005; Andrew et al., 2007). We predicted the miRNA binding sites of functional LncRNAs that may be related to skeletal muscle development. Using a double luciferase test, we found a recognition site of *bta-mir-133a* in the sequence of *LOC104975788* and a binding site for *bta-mir-133a* in the downstream target gene *Pax7*. We speculate that *LOC104975788* may be involved in the regulation of skeletal muscle development by competitively inhibiting the expression of the target gene *Pax7* through binding of *bta-miR-133a*. Future work should test this proposed regulatory mechanism.

## Conclusion

The expression patterns of LncRNAs in skeletal muscle of Shandong black cattle and Luxi cattle were elucidated by RNA sequence analysis, and the LncRNAs that may be involved in the skeletal muscle development of beef cattle were identified. The results allowed construction of interaction networks of LncRNAs-miRNA-mRNA regulated by muscle biology. We speculate that LOC104975788 may be involved in the regulation of skeletal muscle development by competitively inhibiting the expression of the target gene Pax7 through binding of bta-miR-133a.

## Data Availability

The datasets generated during and/or analysed during the current study are available from the corresponding author on reasonable request. The RNA sequencing data has been uploaded to the NCBI GEO database, and can be retrieved using the following accession numbers: GSM4904154, GSM4904155, 623 GSM4904156, GSM4904157, GSM4904158, GSM4904159.

## Abbreviations

DEGs, differentially expressed genes; KEGG, Kyoto Encyclopedia of Genes and Genomes; GO, The Gene Ontology; LncRNA, Long noncoding RNA; miRNA, MicroRNA (miRNA) is a class of noncoding single stranded RNA molecules with a length of about 22 nucleotides encoded by endogenous genes; mRNA, Messenger RNA (mRNA) that encodes proteins.
